# Clinical and immunologic correlates of response to PD-1 blockade in a patient with metastatic renal medullary carcinoma

**DOI:** 10.1186/s40425-016-0206-1

**Published:** 2017-01-17

**Authors:** Kathryn E. Beckermann, Pradeep C. Jolly, Ju Y. Kim, Jennifer Bordeaux, Igor Puzanov, W. Kimryn Rathmell, Douglas B. Johnson

**Affiliations:** 1 Department of Medicine, Division of Hematology/Oncology, Vanderbilt University Medical Center, Nashville, TN 37232 USA; 2 Georgia Cancer Specialists, Atlanta, GA 30342 USA; 3 Genoptix Inc, Carlsbad, CA 92008 USA; 4 Roswell Park Cancer Institute, Buffalo, NY 14263 USA; 5 Vanderbilt-Ingram Cancer Center, 777 Preston Research Building, 2220 Pierce Avenue, Nashville, TN 37232 USA

**Keywords:** Nivolumab, PD-1, Renal medullary carcinoma, Renal cell carcinoma

## Abstract

**Background:**

Renal medullary carcinoma (RMC) is a rare kidney tumor that occurs in adolescent and young adults, typically in association with sickle cell trait. RMC exhibits rapid disease progression, frequent metastases at diagnosis, and dismal clinical outcomes. Currently available therapies, including cisplatin-based combination chemotherapy, multi-tyrosine kinase, and mTOR inhibitor strategies demonstrate either transient responses or minimal activity. Therefore, further molecular characterization and additional treatment strategies are urgently needed in this aggressive disease. The role of immune system surveillance and responsiveness to anti-PD-1 therapies in RMC are completely unexplored.

**Case presentation:**

A 29 year old male with sickle cell trait presented with painless hematuria that ultimately resulted in a diagnosis of RMC. He underwent total nephrectomy and adjuvant cytotoxic chemotherapy with carboplatin, gemcitabine, paclitaxel, and bevacizumab. As is common in this aggressive form of kidney cancer he recurred with biopsy proven lymph node metastasis. He was started on checkpoint inhibitor therapy with nivolumab that inhibits program cell death protein 1 (PD-1), and on his first follow-up imaging he was found to have a partial response that on subsequent scans ultimately resulted in a complete response lasting greater than nine months. In this report, we present a patient with metastatic RMC who exhibited a clinical response to nivolumab, as well as the genetic and immunologic correlates of the pre-treatment tumor. Provocatively, robust immune infiltrate and expression of immune checkpoints were observed, despite the presence of a low mutation burden.

**Conclusions:**

Here, we report the first case of immune microenvironment profiling and response to anti-PD-1 in a patient with RMC to our knowledge. This case suggests that anti-PD-1 based therapies may have clinical activity in RMC.

## Background

Renal medullary carcinoma (RMC) is a rare cancer often referred to as the “seventh sickle cell nephropathy” [1]. The typical patient with RMC is an adolescent or young adult (<40 years old) with sickle cell trait, although other hemoglobinopathies and patients with sickle cell disease have also been encountered [2]. Presenting symptoms include gross hematuria, flank pain, weight loss, fatigue, and often symptoms of metastatic disease. Imaging at diagnosis demonstrates mass lesions centered in the renal medulla, frequently with associated satellite lesions and metastatic spread. The majority of patients present with advanced disease at diagnosis, and RMC has a rapid natural progression of disease with a mean life span with nephrectomy of 16 months [3, 4].

The biology of RMC is only recently being elucidated. The most common genetic event that has been observed is loss of *SMARCB1*/*INI1*. This was first recognized in 2008 based on loss of protein expression [5], and genomic analysis revealed allelic loss and inactivation [6]. More recently, balanced translocations in the tumor suppressor *SMARCB1* have been observed as a cause for this loss [7]. Notably there has been a lack of additional genetic alterations and overall these tumors display relative genome stability [7]. The functional implications of losing this key Swi/Snf complex member remain uncertain. Importantly, the immunologic characteristics of this tumor type have not been thoroughly examined, although one preliminary study suggests that many RMC tumors express PD-L1 [8]. In particular, the profile of tumor infiltrating lymphocytes, immune checkpoint expression, and response to anti-PD-1/PD-L1 have not been described.

Due the rarity of RMC and its rapid clinical progression, therapeutic choices are informed by case reports and small patient series rather than randomized clinical trials. Several reports have shown that patients with RMC can transiently respond to combination platinum-based cytotoxic chemotherapy, but experience little benefit when treated with VEGF or mTOR inhibitors, agents that are conventionally used for clear cell renal cell carcinoma [9–13]. Thus, better therapies for this disease are urgently needed. Here, we report a clinical response of a patient with RMC to PD-1 inhibition, and survey the immunologic characteristics of the pre-treatment tumor.

## Case presentation

A 29 year old previously healthy African American male with sickle cell trait sought evaluation for new onset hematuria. Computed tomography (CT) imaging showed a lesion at the pole of his left kidney and aortocaval lymphadenopathy; a biopsy was consistent with renal medullary carcinoma. He underwent an open left partial nephrectomy with retroperitoneal lymph node dissection. Surgical pathology showed a 2.2 cm tumor with positive margins, Fuhrman nuclear grade 4, involvement of the renal pelvis, and lymphatic invasion with an associated mass of lymph nodes measuring 3 cm in diameter (AJCC stage III – pT1aN1Mx). Next generation sequencing identified only two genomic alterations of known significance, including the loss of SMARCB1 as a homozyogous copy number deletion, and a frame shift mutation in ARID2 (H1495fs*8).

**Fig. 1:**
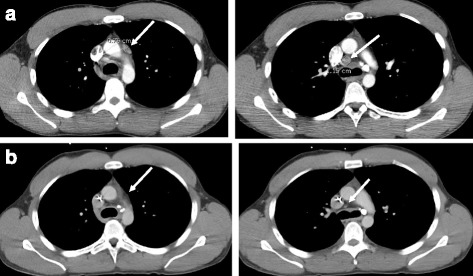
Response to PD-1 inhibition in patient with renal medullary carcinoma. **a** CT chest with contrast showing mediastinal lymphadenopathy prior to treatment with nivolumab (**b**) CT chest with contrast showing resolution of mediastinal lymphadenopathy after nine months on treatment with nivolumab

The patient started an adjuvant treatment regimen of carboplatin AUC of 4, gemcitabine at 1000 mg/m2, and paclitaxel at 80 mg/m2 with the addition of bevacizumab. He experienced significant cytopenias requiring 20% dose reduction to complete 6 cycles of therapy, after which he had no evidence of disease based on imaging. He proceeded with interval cross-sectional imaging every 3 months and had enlargement of a para-aortic lymph node consistent with disease recurrence within 7 months of completing chemotherapy. The patient was reluctant to resume chemotherapy at the time of recurrence due to his prior difficulties with cytopenias and was offered localized therapy with intensity-modulated radiation therapy followed by radiosurgical boost. At completion of his radiotherapy, PET imaging showed resolution of the para-aortic lymph node targeted by radiation, but multiple new enlarged sub-carinal and mediastinal lymph nodes (Fig. 1a).

He then underwent flexible bronchoscopy and biopsy of a right paratracheal lymph node that confirmed disease recurrence. He was initiated on treatment with nivolumab, a PD-1 inhibitor, at 3 mg/kg every two weeks. On his initial CT scan, he was found to have an excellent partial response. At the time of this report, the patient has continued to tolerate nivolumab well with only mild fatigue, and has worked full-time throughout treatment. Now nine months after initiation of PD-1 inhibition, his CT scan showed complete response (Fig. 1b) almost 32 months from the time of his initial diagnosis.

**Fig. 2:**
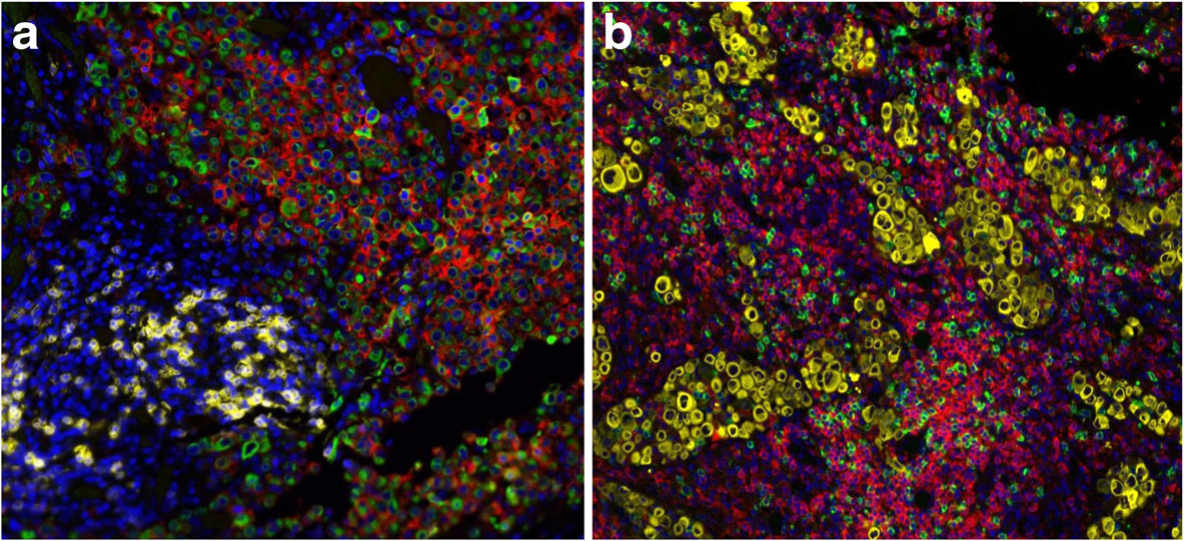
Immunologic correlates by automated quantitative analysis. **a** 20x, DAPI in blue, cytokeratin in green, PD-1 in yellow, and PD-L1 in red. **b** 20x, DAPI blue, cytokeratin yellow, CD8 green, and CD4 red

To elucidate the mechanisms and immune correlates of his response to therapy, we obtained archival tissue from his metastatic, pre-treatment biopsy of the subcarinal lymph node. We performed fluorescent immunohistochemistry using automated quantitative analysis (AQUA®; Genoptix, Inc) to profile the tumor microenvironment. This assay demonstrated substantial expression of PD-L1 (E1L3N antibody) by 23% of tumor cells, and modest expression of both PD-1 and PD-L1 by stromal components (2% and 5% of cells respectively; Fig. 2). Robust expression of both CD8 (10% stromal expression) and CD4 (41% stromal expression) were also demonstrated among infiltrating lymphocytes. Notably, a high percentage of T cells were also positive for CTLA4 (31%). Total infiltrating T cells comprised 21% of all cells in the sample. Interestingly, next generation sequencing of 315 genes (FoundationOne®, Foundation Medicine) showed an extremely overall low mutational burden (projected as <1 mutation/MB).

## Conclusions

Here, we report the first case of immune microenvironment profiling and response to anti-PD-1 in a patient with RMC to our knowledge. RMC is a rare subtype of kidney cancer most commonly diagnosed at advanced stage with a poor overall survival measured in months. We present a unique case of a patient diagnosed with locally advanced disease who underwent surgical resection followed by adjuvant chemotherapy with subsequent rapid disease progression. Radiation to the site of progression was locally effective but he developed simultaneous metastatic spread to multiple mediastinal lymph nodes thereafter. He initiated treatment with the PD-1 inhibitor nivolumab resulting in complete response which is ongoing 9 months into therapy. To our knowledge, this is the first characterization of immune profiling of RMC, and the first case of response to anti-PD-1 in this disease state.

Nivolumab, a monoclonal antibody inhibiting PD-1, was recently approved by the FDA as second line treatment for patients with advanced renal cell carcinoma (RCC). PD-1 is a cell surface signaling receptor found on chronically stimulated T cells which dampens their response and provides negative feedback inhibition. Nivolumab produced a 25% overall response rate compared to 5% with the mTOR inhibitor everolimus in patients with metastatic RCC, thus becoming one of few approved drugs to extend overall survival in this disease (in this case by 5.4 months) [14]. Patients on this and other immunotherapy clinical trials, however, have primarily included the clear cell subtype; RMC is a rare enough entity to not have been studied in this arena to date. There are now numerous ongoing clinical trials using checkpoint inhibitors targeting PD-1 and its ligand PD-L1 in new settings including as upfront treatment, in combination with other checkpoint inhibitors, and in a rapidly expanding range of malignancies.

Numerous efforts to identify markers that may predict response to anti-PD-1 are ongoing, including total numbers of somatic mutations and markers of immune infiltrate (particularly CD8, PD-1, PD-L1, and CTLA-4). High overall mutational burden in various tumor types including colon cancer with mismatch repair deficiency, non-small cell lung cancer, urothelial bladder cancer, and melanoma has also been suggested as a predictive biomarker of response to checkpoint inhibition [15–18]. By contrast, whole exome sequencing and next generation gene panel approaches have found that RMC tumors are genomically quite stable. One could speculate that epigenetic modification, stemming from loss of *SMARCB1*, leads to presentation of neoantigens triggering responses to PD-1 inhibition [19–21]. Mutations in *ARID2* like that found in our patient, have been idenitified to occur in up to 10% of papillary renal cell carcinoma [22]. Interestingly, *ARID2* is also a member of the SWI/SNF chromatin remodeling complex which suggests that loss of function of this complex is important in the pathophysiology of RMC and could lead to enhanced presentation of neoantigens. Notably, our patient was found to have a vigorous immune infiltrate including CD4+ and CD8+ T cells and high levels of immune checkpoint expression, including PD-L1, CTLA-4, and PD-1, which suggest pre-existing immune recognition, and have also been linked to immune therapy responses [23–25]. By contrast, the patient was found to have a very low calculated mutation burden using targeted next generation sequencing. Thus, this study suggests alternative paradigms of immune responsiveness beyond mutational load.

In an adolescent and young adult disease such as RMC where median overall survival is 16 months and current treatment options are suboptimal, additional biologic insights and novel therapeutics are urgently needed. This case report suggests a potential role for PD-1/PD-L1 inhibition in RMC. Further investigation in RMC and other rare RCC subtypes is necessary to determine the benefit of immune checkpoint inhibitors in patients with various subtypes of kidney cancer.

## Abbreviations

(AJCC): American joint comission on cancer
(AUC): Area under the curve
(CT): Computed tomography
(CTLA-4): Cytotoxic T-lymphocyte associated protein 4
(FDA): Food and drug administration
(mTOR): Mammalian target of rapamycin
(PD-1): Progammed cell death 1 protein
(PD-L1): Programmed death ligand-1
(PET): Positron emission tomography
(RCC): Renal cell carcinoma
(RMC): Renal medullary carcinoma
(SMARCB1): SWI/SNF Related matrix associated actin dependent regulator of chromatin
